# Health Perceptions and Trust in Healthcare After COVID-19: An Exploratory Cross-Sectional Survey from Romania

**DOI:** 10.3390/ijerph22101496

**Published:** 2025-09-27

**Authors:** Réka Bodea, Alexandra Maria Buboacă, Lorand Iozsef Ferencz, Zoltán Ábrám, Toader Septimiu Voidăzan

**Affiliations:** 1 Department of Epidemiology, George Emil Palade University of Medicine, Pharmacy, Science, and Technology of Targu Mureș, 540142 Târgu Mureș, Romania; septimiu.voidazan@umfst.ro; 2 George Emil Palade University of Medicine, Pharmacy, Science, and Technology of Targu Mureș, 540142 Târgu Mureș, Romania; 3 Department of Hygiene, George Emil Palade University of Medicine, Pharmacy, Science, and Technology of Targu Mureș, 540142 Târgu Mureș, Romania; lorand.ferencz@umfst.ro (L.I.F.); zoltan.abram@umfst.ro (Z.Á.)

**Keywords:** COVID-19, health knowledge, attitudes, practice, trust, health services accessibility, cross-sectional studies, Romania

## Abstract

Background: This study is particularly relevant to the Romanian context, where relatively few empirical investigations have examined post-pandemic health perceptions and levels of trust in public institutions. The purpose of this study is to investigate the long-term impact of the COVID-19 pandemic on health perceptions and trust in the healthcare system by examining key socioeconomic and epidemiological factors. Methods: A cross-sectional online survey was conducted among Romanian adults (*N* = 423), between March and April 2025. Demographic data, lifestyle habits, mental health, and access to healthcare were assessed. Statistical analyses included both bivariate (chi-square test) and multivariable logistic regression models to identify independent associations. Results: 31.9% of participants reported increased stress and anxiety during the pandemic. Decreased trust in the healthcare system (75.6%) and a perceived reduction in life expectancy (74.3%) were also noted as a consequence of the COVID-19 pandemic. Perceived life expectancy decline was linked to lower education and inconsistent healthcare behavior. Conclusion: In our sample, the perception of decreased life expectancy reflects not only epidemiological realities but also emotional and social responses to crises. Individuals’ trust, behavior, and shared vision of the future have also been challenged during the COVID-19 pandemic.

## 1. Introduction

Life expectancy (LE) is a key macroeconomic indicator of a population’s health and social development. LE reflects the performance of the health system, as well as the mental, economic, and structural features of society [1]. Data from the World Health Organization (WHO) and Eurostat indicate that LE in Romania increased continuously between 2015 and 2019 (reaching 75.5 years in 2019). The COVID-19 pandemic, with its direct and indirect effects, dramatically interrupted this trend. By 2021, LE at birth in Romania had decreased by 2.8 years, one of the largest decreases in the European Union [2]. Based on data from 21 industrialized countries, in 2020 Kontis et al. reported more than 200,000 excess deaths in the first wave, corresponding to sharp declines in LE across Europe, particularly in Spain, England, and Wales, and Italy [3]. At the global level, Heuveline estimated that LE fell by 0.92 years in 2020 and an additional 0.72 years in 2021, representing the first global decline since 1950 [4]. These findings underscore the magnitude of the mortality shock associated with the pandemic.

Direct (hospitalization, mortality, and post-COVID syndrome) and indirect effects, such as a lack of care, diagnostic delays, mental health problems, and distrust in the health system, have been supported by studies [3,5,6]. The indirect impact of the pandemic has been substantial and, in many cases, more difficult to measure than the direct effects. Restrictions on mobility and the temporary closure of businesses have hit both developed and developing economies. On the social front, isolation, the rapid shift to online education, working from home, and general uncertainty have added additional burdens—on mental health, access to services and community cohesion [7]. Research from 2020 to 2022 indicated a notable growth in the levels of stress, anxiety, and depressive symptoms in the general population, especially among young people and those living in urban areas [8,9]. In parallel, another study showed a decline in health system trust, accompanied by a decrease in adherence to treatment and poorer perceived health outcomes [10]. In Europe, Eurofound’s “Living, working and COVID-19” survey documented how the pandemic not only influenced health outcomes but also shaped public perceptions of quality of life and trust in institutions, uncovering substantial disparities between countries [11]. Trust has been acknowledged as an important factor in guiding both compliance and public health resilience. The implementation of government measures and public adherence to restrictions was driven by political and social trust, a fact concluded by Devine et al. in early stages of the pandemic [12]. Corroborating evidence from Pak et al., based on data from more than 100,000 individuals in 58 countries, showed that higher levels of trust doubled the impact of severe restrictions on compliance [13].

The evolution of the COVID-19 pandemic in Romania has been similar to global trends. According to official reports from Romania, 3,504,870 cases and 68,611 deaths have been registered [14]. Romania experienced several pandemic waves between March 2020 and the end of 2022. The first wave (March-May 2020) was well managed through the implementation of strict isolation measures, the enforcement of curfews, and the suspension of social and economic activities. The second wave (October–December 2020) began in the context of premature relaxation of restrictions, characterized by a sharp increase in the number of cases and deaths. The third (February–April 2021) coincided with the emergence of the Alpha variant, while the fourth (August–November 2021), dominated by the Delta variant, was the most serious in terms of mortality, with Romania having one of the highest death rates in the world per capita [15,16]. The fifth wave (January–March 2022), associated with Omicron BA.1, resulted in a record number of daily cases, accompanied by fewer hospitalizations and deaths. Subsequently, the sixth wave (summer–autumn 2022) was determined by the Omicron BA.5 and BQ.1 subvariants, in a context of relaxation of most health measures and higher vaccination coverage [17,18].

In Romania, the pandemic’s impact was exacerbated by several factors. An important health migration occurred, resulting in a shortage of doctors in rural areas. The healthcare system had structural deficiencies, and additionally, the population lacked trust in institutions, which contributed to low vaccination coverage (approximately 40–45% in 2021). The high prevalence of chronic diseases (hypertension, diabetes, and chronic obstructive pulmonary disease), along with low levels of health awareness and preventive involvement [19,20,21], is a major concern. These factors should be emphasized when assessing the pandemic’s impact in Romania.

While international research has largely documented the associated mental health burden and loss of trust in healthcare, there is still a lack of knowledge on how these were perceived at the population level in Romania. Previous studies have mostly focused on mortality, vaccination, or healthcare utilization, but subjective perceptions of LE and institutional trust remain underexplored. This scientific gap is particularly relevant in Central and Eastern Europe, where structural weaknesses of the healthcare system and low vaccination uptake may have amplified the psychosocial impact of the pandemic.

Our study aims to fill the gap and investigate how the pandemic has influenced public perceptions. The specific objectives were to examine (i) subjective perceptions of life expectancy decrease, (ii) psychological distress during the pandemic, and (iii) levels of trust in the healthcare system. Based on previous reports, documenting both the objective decline of LE in Romania (Eurostat, WHO) and international evidence of increased stress, anxiety, and reduced trust in healthcare during the COVID-19 pandemic, we hypothesized that the pandemic in Romania not only reduced LE in statistical terms, but also generated a subjective perception of decreased LE, accompanied by higher levels of psychological distress and a decline in institutional trust.

## 2. Materials and Methods

### 2.1. Study Design

Our study was quantitative, cross-sectional, descriptive, and analytical in nature. A survey was conducted between March and April 2025. Romanian citizens were surveyed.

### 2.2. Sample Size and Sampling Method

The sample size was calculated using Cochran’s formula for a large population (95% confidence level, 5% margin of error, *p* = 0.5), resulting in *N* = 385. This calculation was confirmed using the Raosoft Sample Size Calculator (Raosoft, Inc., Seattle, WA, USA). To ensure sufficient precision for subgroup analyses (e.g., sex, age, region), we planned a ~10% oversampling. Participants were selected through convenience sampling, and the questionnaire was distributed via various digital platforms (including email, social media networks, and messaging applications). With a total of 423 respondents, the study achieved >95% statistical power to detect medium effect sizes (Cohen’s w = 0.3) at α = 0.05.

### 2.3. Data Collection

A Romanian electronic survey was created using Google Forms. The online questionnaire (full version available in Supplementary File S1) consisted of 37 closed- and open-ended questions, with the following content blocks: demographic data (age, sex, education, occupation, and place of residence); lifestyle and health status (chronic diseases and SARS-CoV-2 infection); mental health (causes of death among relatives, stress, anxiety, and depression); access to health care; confidence in the health system and self-perception of LE. No formal definitions were provided for subjective concepts such as “healthy diet” or “active lifestyle”, and participants interpreted these terms according to their own understanding. The questionnaire did not include an instruction guide, which may have contributed to variability in interpretation. This choice was intentional to capture participants’ personal interpretations, which are part of the subjective perception under study. The questionnaire was validated by peer review (face validity) and pilot testing (*N* = 15), after which stylistic refinements were made. Inclusion criteria were Romanian citizenship, age ≥ 18 years, provision of informed consent, and complete submission of the questionnaire. Exclusion criteria were incomplete questionnaires (automatically filtered by Google Forms). Because all items in the survey were mandatory, no missing data were present in the final dataset. We recognize that the absence of a detailed instruction guide may have increased variability in responses; however, this variability mirrors real-world differences in how individuals perceive and report health-related concepts. Accordingly, the questionnaire was designed to preserve methodological rigor while maintaining ecological validity. This allowed it to capture authentic, everyday perspectives without sacrificing scientific standards. Age was recorded directly in predefined categories (<20, 20–30, 31–45, 46–65, >65), as structured in the original questionnaire. These cut-offs were chosen a priori, anticipating that the 20–30 age group would represent the largest share of online respondents, while the 65+ group was retained as a distinct category due to its epidemiological relevance. Other quantitative variables (e.g., frequency of medical check-ups) were also collected in predefined ordinal categories, while binary questions (e.g., smoking, active lifestyle, trust in healthcare) were treated as dichotomous variables.

### 2.4. Data Processing and Statistical Analysis

The data were downloaded and prepared (coded) in Microsoft Excel and then imported into the Statistical Package for the Social Sciences (SPSS, version 23; IBM SPSS Statistics for Windows, Armonk, NY, USA). Data analysis was conducted in a structured, integrated framework consisting of three main stages. Descriptive statistics were first performed to provide an overview of the study population. Frequencies and percentages were calculated for categorical variables, while means with standard deviations were used for continuous measures. This step allowed us to characterize the sociodemographic, behavioral, and health-related profile of respondents. Bivariate analysis was then carried out to explore preliminary associations between associated factors and outcomes. Cross-tabulations were generated, and Pearson’s chi-square (χ^2^) test was used to assess relationships between categorical variables. When expected cell counts were below assumptions, Fisher’s exact test was applied. A significance threshold of *p* < 0.05 was used. This stage identified potential determinants of three outcomes (1—increased stress and anxiety during the pandemic; 2—decreased trust in the healthcare system; 3—perceived decrease in life expectancy) and provided the basis for multivariable modeling. Multivariable logistic regression models were finally applied to examine the independent contribution of each variable while controlling for potential confounding factors. Separate models were built for each outcome. Variables included in the models were those statistically significant in the bivariate analysis or deemed conceptually important. Results were expressed as adjusted odds ratios (OR) with 95% confidence intervals (CI) and corresponding *p*-values. Only associated factors that remained significant were retained in the final tables. For the regression analysis, age and education were included as categorical variables, consistent with their predefined categories in the questionnaire, to facilitate interpretation, comparability with demographic statistics, and to ensure sufficient group sizes for analysis. Model assumptions were verified: all predictors were categorical or binary, so the assumption of logit linearity did not apply; multicollinearity was tested using Variance Inflation Factor (VIF), with all values < 2; and model fit was assessed with the Hosmer–Lemeshow goodness-of-fit test, which indicated adequate fit (*p* > 0.05).

## 3. Results

### 3.1. Sample Description—Sociodemographic Profile

A total of 423 fully completed questionnaires were included in the analysis. The inclusion process is illustrated in Supplementary Figure S1. In total, 80.8% of the respondents were women, with 73% of respondents aged between 20 and 30 years old. Furthermore, 69.9% of the participants lived in rural areas. The majority had secondary education: 69.5% had completed secondary school, 21.5% had completed university, and 5.2% had completed postgraduate education. A total of 59.8% were students, among those employed (*N* = 118), 29 were healthcare workers (Table 1).

### 3.2. Health Behavior Patterns

A total of 367 (86.7%) reported an active lifestyle, 20 (4.5%) were smokers, and 14 (3.3%) were exposed to toxic environments. In total, 360 (85.1%) participants reported eating a healthy and balanced diet. 343 (81.1%) visited their general practitioner (GP) or specialist when experiencing health complaints. A total of 244 (57.7%) people had very infrequent medical check-ups, while 33 (7.8%) people had two check-ups per year; only 122 (28.8%) had routine annual check-ups. Furthermore, 294 (69.5%) participants reported regularly keeping their medical and screening appointments (Table 2).

38.5% reported being infected with SARS-CoV-2. Among these participants, 56.4% had mild symptoms, 38.6% had moderate symptoms, and only 5.9% had severe symptoms. Just 1.8% required hospitalization.

### 3.3. Indirect Impact of the Pandemic

**Figure 1:**
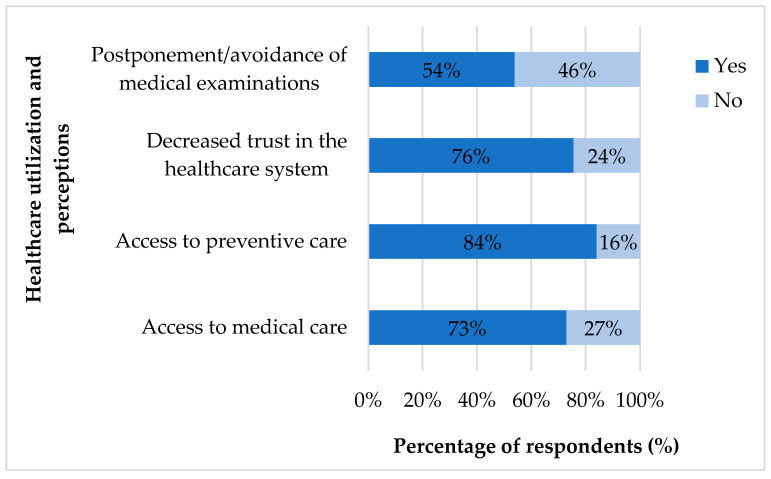
The Impact of the pandemic on healthcare utilization and perceptions.

We inquired about access to healthcare at that time to obtain a comprehensive picture of the pandemic’s impact. Here, 309 (73%) people reported experiencing difficulties accessing specialists or primary care. Moreover, 356 (84.1%) experienced a decrease in access to prevention services and screening. Additionally, 320 (75.6%) reported a decrease in trust in the health system. Furthermore, 228 (53.9%) respondents reported social withdrawal, postponing, or avoiding medical check-ups during the epidemic (Figure 1). During the pandemic, they obtained information on health-related issues primarily from digital sources (e.g., the internet or health portals), doctors, and their close social circle, such as family members or acquaintances.

In total, 31.9% of the respondents said that they experienced increased levels of stress or anxiety during the pandemic. Furthermore, 79.9% reported symptoms of depression or anxiety in themselves or people close to them. Three-quarters of the respondents (74.2%) believed that LE in Romania had decreased as a consequence of the COVID-19 pandemic. According to their subjective experience, the prevalence of chronic diseases had also increased, contributing to this perceived decline in LE, with 75.4% agreeing with this hypothesis. In response to an open-ended question, nearly half of the respondents stressed that a healthy lifestyle, stress reduction, and prevention are key to a longer life. In total, 24.6% believed that vaccination against COVID-19 is an effective way to reduce the risk of disease. There was an increase in health awareness (73.5%), despite a significant lack of confidence. Furthermore, 82.0% said that the pandemic had led to increased interest in public health and epidemiological research.

### 3.4. Association Between Sociodemographic and Behavioral Factors and Perceived Outcomes

The analysis was performed in two stages. First, bivariate associations between the three primary outcome variables (increased stress during the pandemic, reduced trust in the healthcare system, and perceived decrease in LE) and selected sociodemographic and behavioral variables were tested using chi-square tests. The results are presented in Table 3 and Table 4.

Subsequently, multivariable logistic regression models were conducted to identify independent factors for each outcome (Table 5). Only those variables that showed statistical significance in bivariate analyses or were conceptually relevant were included. The models provided adjusted OR with 95% CI.

Overall, the multivariable models confirmed several associations identified in the bivariate stage while controlling for potential confounders. Stress and anxiety were influenced by both sociodemographic and behavioral variables. In contrast, trust in healthcare and perceived decrease in LE were more closely linked to age, education level, and preventive behaviors.

Supplementary Material Table S1 shows that gender was significantly associated with medical appointment adherence, symptoms of depression/anxiety, and decreased trust in the healthcare system. Age was associated with a healthy diet and reduced stress/anxiety during the pandemic. At the same time, residence was linked to postponement of medical examinations, perceived decrease in LE, and increased interest in public health. Education level was associated with difficulties in accessing care, reduced preventive services, stress/anxiety, and perceived decrease in LE (all *p* < 0.05).

## 4. Discussion

On a sample of 423 Romanian adults, we examined the indirect impacts of the COVID-19 pandemic. In addition to assessing participants’ perceptions, we explored lifestyle risks, mental health, access to health services, and loss of trust in health care. These factors ultimately contributed to the perception of a decline in LE. Based on modelling by Aburto et al., 95% of the reduction in LE between 2020 and 2021 was attributed to COVID-19-related deaths [22]. In our study, 74.2% of participants reported a subjective perception of decreased LE, which was consistent with the official Eurostat estimate [2]. A greater proportion of rural respondents perceived that LE had decreased (Residence ↔ Perceived decreased LE; *p* = 0.0001), which may reflect structural issues in rural healthcare.

### 4.1. Lifestyle Risks

Although a large proportion of respondents reported health-conscious behavior (86.7% reported being active, and 85.1% reported eating healthily), actual health prevention behavior remained low. In our sample, older age groups were more aware of their diet, whereas young adults were less aware (Age group ↔ Healthy eating; *p* = 0.0001). This also suggests that the target group for health education is 20–30-year-olds, who require the most focused interventions. In total, 28.8% of the respondents attended annual check-ups, while more than half rarely attended them. Women were significantly more likely to have regular check-ups than men (No ↔ Regular medical check-ups; *p* = 0.007). This means that women are more health-conscious, but men are less likely to receive a timely diagnosis. Among behavioral factors, regular use of medical care (check-ups and adherence) was most strongly associated with all three pandemic consequences: stress, loss of confidence, and the perception of decreased LE. Conscious health behavior influences not only objective but also subjective health experiences.

According to the European Observatory on Health Systems and Policies’ National Health Profile 2021, Romania has the second-lowest per capita expenditure on preventive health in the EU. From 2018 to 2019, new screening programs were launched; however, the population’s participation continued to decline [23]. According to the 2022 Health Policy Report, Romania experienced a 47% decrease in preventive screening and timely diagnosis between 2020 and 2021, especially in rural regions [19]. Poor participation in screening and health culture, especially in rural and low-income groups, carries significant risks for both individual and public health outcomes. Thus, in the 2023 report, participation rates remained below the European average (12–30% for cervical cancer screening and 33% for mammography screening) [24]. A Romanian pilot study revealed a significant decrease in colorectal screening during the COVID-19 pandemic, although improvements were later observed [25].

This behavioral dissonance (healthy living and not seeking medical screening) can be explained by Ajzen’s theory of planned behavior (TPB). In public health, many campaigns assume that if people know something, they will act on it. The TPB indicates that attitudes alone are insufficient if there is no support, access, trust, or positive social expectation [26]. In a Dutch study, the number of cancer diagnoses markedly decreased during the COVID-19 pandemic, suggesting a disruption in preventive screening services [27].

Lifestyle factors have a long-term detrimental effect on health status, thereby indirectly reducing LE. This justifies a rethinking of health behavior and preventive health services. Institutional-level support could shift some of the responsibility, for example, through targeted health education programs (especially among young adults and people living in rural areas), automated screening recalls, or community prevention campaigns.

### 4.2. Mental Health Outcomes

Mental health deterioration (increased anxiety, depression) has been widely studied and documented. Romanian studies have shown similar levels of mental distress (especially among young adults and women) [28,29]. According to our questionnaire, these two groups were also the most affected (gender ↔ depression/anxiety symptoms among close contacts; *p* = 0.0001). Women were more affected by mental strain, probably also due to the increased presence of caregiving roles and social sensitivity. Psychological distress was greater among 20–30-year-olds (Age group ↔ Increased stress and anxiety during the pandemic; *p* = 0.0004). The vulnerability and uncertainty about the future (career start, finances) of young adults explain their increased emotional reactions. Low educational attainment was significantly associated with increased mental distress (educational attainment ↔ Increased stress and anxiety; *p* = 0.0001). Financial insecurity, a lack of prospects, and job loss increased distress. A significant finding was that people with lower medical appointment adherence had a higher likelihood of both stress/anxiety and perceiving a decline in life expectancy. A longitudinal cohort study showed that anxiety was a significant factor behind missed medical visits. This reveals that missed care was not just a pragmatic choice but was primarily motivated by psychological factors [30].

The existence of a “second wave of the pandemic”, namely mental health consequences, was demonstrated by researchers quite early on [31]. Deterioration in mental health is associated with mortality, as several studies have shown. Mental health deterioration (particularly depression and anxiety) significantly increases the risk of mortality, both directly (e.g., suicide) and indirectly (e.g., avoided care, exacerbation of chronic illness) [32]. Mental health is critical issue worldwide. Global responses remain inadequate. The WHO, in its “World Mental Health Report: Transforming Mental Health for All”, calls on all stakeholders to work together to strengthen systems in a committed way [33].

### 4.3. Trust Loss and Access to Health Care

In total, 75.6% of respondents reported a decrease in trust in the health system, while 53.9% had postponed or avoided medical appointments. Women were more likely to have lost trust in the health care system (No ↔ Decreased trust in health care; *p* = 0.0251). This is particularly important, as women are more frequent users of health care; thus, loss of trust can have more serious consequences for them (e.g., such as postponing medical care for children). This combination, in itself, compromises the functionality of both outpatient and inpatient care. It can be argued that people who have no faith in the health system do not seek care; thus, delayed identification and insufficient management of chronic diseases affect survival rates.

The most vulnerable subgroups are young adults, those with low education levels, and the rural population. In our study, they reported the highest proportions of loss of confidence, barriers to care, and negative perceptions of the future. Rural respondents were more likely to postpone or avoid medical appointments (Residence ↔ Postpone/avoid medical appointments; *p* = 0.006). This suggests structural access inequalities, including fewer services, fewer appointments available, and a less prevalent preventive culture in rural areas.

A representative survey of 3789 people in six Western Balkan countries (Albania, Bosnia and Herzegovina, Kosovo, Northern Macedonia, Montenegro, and Serbia) was used to calculate an average confidence score. The main results of this study show that citizens in these countries have a low level of trust in their health care system (4.3/10), with doctors working in private health care institutions having a trust score of 6.6/10 and those working in the public sector having a level of trust of 5.7/10 [34].

According to the Health Belief Model, perceived vulnerability should motivate preventive behavior, but only when perceived benefits outweigh perceived barriers, and individuals feel capable of taking effective action. During the COVID-19 pandemic, many individuals may have perceived themselves as being at high risk but lacked trust in the system, faced access issues, or doubted their ability to navigate the healthcare process, leading to inconsistent health behaviors. This inconsistency in turn likely increased feelings of insecurity and perceived vulnerability [35,36].

The loss of trust and avoidance of medical examinations after the pandemic had serious public health consequences. Empathetic doctor–patient communication, the involvement of local health mediators, and transparent and socially credible health campaigns are needed to restore trust. Additionally, structural strengthening of the healthcare system in Romania structurally and improving accessibility, particularly in rural areas, are crucial.

### 4.4. Study Limitations

This study has several limitations. First, like all cross-sectional studies, the present study has a limited ability to draw causal relationships. Temporal sequencing between exposures and outcomes cannot be established. Second, the sampling method was convenience-based and online; therefore, the sample is not representative, and disadvantaged groups may be underrepresented due to the lack of digital access. In particular, younger, female, and rural respondents were overrepresented in our sample, which may limit the generalizability of the results to the national population. The overrepresentation of young women in our sample may have inflated the reported levels of psychological distress compared to the general population. Third, data collection relied on self-reports, which may have introduced response bias. The absence of formal definitions for certain subjective concepts (e.g., “healthy diet”, “active lifestyle”) and the lack of a detailed instruction guide may have introduced variability in interpretation, although this was intentional to capture authentic subjective perceptions. However, the level of detail in the questionnaire, the sample size (*N* = 423), and the significant statistical results allowed for reliable and meaningful trends to be identified. The results should be interpreted as indicative of patterns among digitally connected Romanian adults rather than as fully representative at the national level. Digital bias should be considered, as groups with limited internet access (e.g., older adults, lower socioeconomic categories) were less likely to be included. No formal sensitivity analyses were performed, which is a limitation of our study, as we could not test the robustness of associations under alternative model specifications. In addition, as multiple bivariate and multivariable analyses were conducted, the possibility of type I error cannot be excluded. These factors reinforce the need for caution when interpreting the magnitude of associations and further limit the generalizability of our findings. The findings emphasize vulnerable groups and behavioral trends that align with international evidence, offering a valuable starting point for more representative and longitudinal studies.

## 5. Conclusions

This study set out to investigate how the COVID-19 pandemic has affected health perceptions and trust in the healthcare system in Romania, focusing on three main outcomes: perceived decrease in LE, psychological distress, and institutional trust. By employing an integrated analytical framework, we captured not only population-level patterns but also the independent associated factors of these outcomes. According to our findings, the perceived risks to individuals and society during the pandemic were exacerbated by inconsistent health behavior, structural barriers to healthcare access, and decline in institutional trust.

Structural reforms alone are insufficient to achieve a post-pandemic recovery. Rebuilding trust, supporting preventive care services, and enhancing health education—particularly for young people and those living in rural areas—are crucial from a public health perspective. In addition to improving objective health outcomes, closing behavioral and structural gaps can make society better prepared and more health-conscious in times of crisis.

In conclusion, this research contributes to the growing body of evidence on the indirect impacts of COVID-19 by highlighting the interconnectedness of health perceptions, behaviors, and institutional trust.

## Figures and Tables

**Table 1:** Demographic characteristics of the study sample.

Variables	N (%) 423 (100%)
Gender	
Female	342 (80.8%)
Male	81 (19.1%)
Age groups	
<20 years	33 (7.8%)
20–30 years	309 (73.0%)
31–45 years	45 (10.6%)
46–65 years	30 (7.0%)
>65 years	6 (1.4%)
Residence	
Urban	127 (30.0%)
Rural	296 (69.9%)
Education level	
No formal education	5 (1.1%)
High school	294 (69.5%)
Postsecondary nontertiary education	11 (2.6%)
University studies	91 (21.5%)
Postgraduate studies	22 (5.2%)
Occupation	
Student/Pupil	253 (59.8%)
Unemployed/Household	46 (10.8%)
Employed	118 (27.9%)
Retired	6 (1.4%)

**Table 2:** Lifestyle habits of the study sample. GP—general practitioner.

Variables	N (%) 423 (100%)
Smoking	
Yes	20 (4.7%)
No	403 (95.3%)
Toxic environment (workplace/home)	
Yes	14 (3.3%)
No	409 (96.7%)
Active lifestyle	
Yes	367 (86.7%)
No	56 (13.2%)
Healthy diet	
Yes	360 (85.1%)
No	63 (14.9%)
Timely healthcare seeking	
Yes	343 (81.1%)
No	76 (18.0%)
No, I do not have a GP	4 (0.9%)
Medical check-up frequency	
>2x/year	24 (5.7%)
2x/year	33 (7.8%)
1x/year	122 (28.8%)
Rarely	244 (57.7%)
Medical appointment adherence	
Yes	294 (69.5%)
No	129 (30.5%)

**Table 3:** Results of chi-square tests assessing the associations between the three main outcome variables (stress and anxiety, decreased trust in healthcare, and perceived decrease in life expectancy) and key sociodemographic factors. The table reports χ^2^ statistics, degrees of freedom (df), and *p*-values. Abbreviation: LE—life expectancy.

Outcome Variables	Sociodemographic Variables	N (%)	χ^2^	df	*p*-Value
Increased stress & anxiety during the pandemic	Age	423 (100%)	61.84	4	<0.001
	Gender	423 (100%)	6.54	1	0.011
	Education level	423 (100%)	53.41	4	<0.001
	Residence	423 (100%)	49.66	1	<0.001
Decreased trust in the healthcare system following the pandemic	Age	423 (100%)	36.48	4	<0.001
	Gender	423 (100%)	5.01	1	0.025
	Education level	423 (100%)	33.12	4	<0.001
	Residence	423 (100%)	23.41	1	<0.001
Perceived decrease in LE following the pandemic	Age	423 (100%)	49.79	4	0.001
	Gender	423 (100%)	10.79	1	0.001
	Education level	423 (100%)	49.06	4	<0.001
	Residence	423 (100%)	16.55	1	<0.001

**Table 4:** Chi-square test results of associations between the three main outcome variables (stress and anxiety, decreased trust in healthcare, and perceived decrease in life expectancy) and selected behavioral and preventive health variables. The table reports χ^2^ statistics, degrees of freedom (df), and *p*-values. Abbreviation: LE—life expectancy.

Outcome Variables	Behavioral Variables	N (%)	χ^2^	df	*p*-Value
Increased stress & anxiety during the pandemic	Active lifestyle	423 (100%)	36.49	1	<0.001
	Smoking	423 (100%)	12.23	1	0.00
	Toxic environment (workplace/home)	423 (100%)	0.36	1	0.547
	Healthy diet	423 (100%)	75.16	1	<0.001
	Medical check-up frequency	423 (100%)	49.00	3	<0.001
	Medical appointment adherence	423 (100%)	83.49	1	<0.001
Decreased trust in the healthcare system following the pandemic	Active lifestyle	423 (100%)	10.87	1	<0.001
	Smoking	423 (100%)	1.97	1	0.160
	Toxic environment (workplace/home)	423 (100%)	1.75	1	0.185
	Healthy diet	423 (100%)	5.19	1	0.023
	Medical check-up frequency	423 (100%)	44.93	3	<0.001
	Medical appointment adherence	423 (100%)	17.68	1	<0.001
Perceived decrease in LE following the pandemic	Active lifestyle	423 (100%)	0.46	1	0.497
	Smoking	423 (100%)	0.50	1	0.481
	Toxic environment (workplace/home)	423 (100%)	3.23	1	0.072
	Healthy diet	423 (100%)	1.77	1	0.183
	Medical check-up frequency	423 (100%)	11.23	3	0.011
	Medical appointment adherence	423 (100%)	46.56	1	<0.001

**Table 5:** Logistic regression results for stress and anxiety, decreased trust in healthcare, and perceived decrease in life expectancy. Table includes OR, 95% CI, Wald χ^2^ statistics with degrees of freedom, and *p*-values for significant associated factors. Reference categories: Age = 20–30 years; Gender = Female; Residence = Rural; Education = High school; Healthy diet = Yes; Medical appointment adherence = Yes; Ed. level—Education level; Postsec. nontertiary ed.—Postsecondary nontertiary education, LE—life expectancy.

Variable	N (%)	B	SE	Wald χ	df	*p*-Value	OR (ExpB)	95% CI
Predictors of Stress/Anxiety								
Age: 31–45 yo	45 (10.6%)	1.136	0.511	4.935	1	0.026	3.113	[1.143–8.478]
Age: 46–65 yo	30 (7.0%)	1.560	0.530	8.673	1	0.003	4.758	[1.685–13.436]
Age: >65 yo	6 (1.4%)	2.410	0.931	6.698	1	0.009	11.135	[1.795–69.081]
Gender: Male	81 (19.1%)	−0.968	0.373	6.738	1	0.009	0.380	[0.183–0.789]
Residence: Urban	127 (30.0%)	1.023	0.307	11.129	1	0.000	2.781	[1.524–5.072]
Healthy diet: No	63 (14.8%)	1.855	0.441	17.654	1	<0.001	6.389	[2.689–15.176]
Medical appointment adherence: No	129 (30.4%)	1.514	0.313	23.432	1	<0.001	4.543	[2.461–8.385]
Decreased Trust in Healthcare								
Age: <20 yo	33 (7.8%)	−2.079	0.486	18.34	1	0.0	0.125	[0.051–0.306]
Residence: Urban	127 (30.02%)	−0.607	0.302	4.04	1	0.035	0.545	[0.309–0.96]
Perceived Decrease in LE								
Age: <20 yo	33 (7.8%)	−2.313	0.548	17.82	1	<0.001	0.099	[0.038–0.261]
Age: >65 yo	6 (1.4%)	−2.163	0.878	6.08	1	0.014	0.115	[0.019–0.702]
Ed. level: Postsec. nontertiary ed.	11 (2.6%)	−2.554	1.079	5.61	1	0.018	0.078	[0.006–0.974]
Ed. level: University studies	91 (21.5%)	−2.526	0.978	6.67	1	0.010	0.080	[0.009–0.688]
Ed. level: Postgraduate studies	22 (5.2%)	−3.194	1.283	6.20	1	0.013	0.041	[0.004–0.433]
Medical appointment adherence: No	129 (30.4%)	−1.356	0.314	18.68	1	<0.001	0.257	[0.140–0.471]

## Data Availability

Data access was restricted to investigators and authorized personnel.

## Supplementary Materials

The following supporting information can be downloaded at: https://www.mdpi.com/article/10.3390/ijerph22101496/s1, File S1: Full Questionnaire Used in the Study: Health Perceptions and Trust in Healthcare after COVID-19 (Romania, 2025); Figure S1: Flowchart of inclusion process; Table S1: Statistically significant associations between demographic characteristics and pandemic-related outcomes

## Abbreviations

The following abbreviations are used in this manuscript:

χ^2^	Chi-Square
CI	Confidence Interval
COVID-19	Coronavirus Disease 2019
df	Degrees of Freedom
EU	European Union
GP	General Practitioner
LE	Life Expectancy
OR	Odds Ratio
SPSS	Statistical Package for the Social Sciences
VIF	Variance Inflation Factor
TPB	Theory of Planned Behavior
WHO	World Health Organization
