# Supplementary material for: Health Perceptions and Trust in Healthcare After COVID-19: An Exploratory Cross-Sectional Survey from Romania
Source: Int J Environ Res Public Health. 2025 Sep 27;22(10):1496. doi: 10.3390/ijerph22101496 (PMC12564094; doi:10.3390/ijerph22101496)
Supplement: Supplementary file 1 [file ijerph-22-01496-s001.zip › ijerph-3809423_Supplementary File S1.pdf]

No.	Question	Response Options
1	What is your age group?	☐ <20 ☐ 20–30 ☐ 31–45 ☐ 46–65 ☐ >65
2	What is your gender?	☐ Male ☐ Female
3	What is your highest level of education?	☐ No formal education ☐ High school ☐ Postsecondary nontertiary education ☐ University studies<br>☐ Postgraduate studies
4	What is your current occupation?	Open-ended response
5	What is your environment of origin?	☐ Urban ☐ Rural
6	Are you a smoker?	☐ Yes ☐ No
7	Have you been exposed to toxic substances at work or at home?	☐ Yes ☐ No
8	Do you have an active lifestyle?	☐ Yes ☐ No
9	Do you have a balanced and healthy diet?	☐ Yes ☐ No
10	Do you go to your family doctor/specialist early when you have a health problem?	☐ Yes ☐ No ☐ I do not have family doctor
11	How often do you have regular medical check-ups?	☐ More than twice a year<br>☐ Two times a year ☐ Once a year<br>☐ Very rarely
12	Do you keep your regular appointments for medical check-ups and screenings?	☐ Yes ☐ No
13	Have you been diagnosed with a chronic illness?	☐ Yes ☐ No
14	What chronic conditions do you have? (If you do not have one, just write "no")	Open-ended response
15	Have you had COVID-19?	☐ Yes ☐ No
16	If you were infected with COVID-19, what was the severity of the symptoms?	☐ Mild ☐ Moderate ☐ Severe<br>☐ I did not had COVID-19
17	If yes, were you hospitalized during the infection?	☐ Yes ☐ No<br>☐ I did not have COVID-19
18	Do you have first/second degree relatives who died during the pandemic?	☐ Yes ☐ No
19	If you have relatives who died during the pandemic, what pathology (disease) did they die from? (If you don't, just write "no"):	Open-ended response
20	Do you know people who died most often during the pandemic from:	☐ COVID-19 ☐ Cancer<br>☐ Cardiovascular disease ☐ Respiratory disease ☐ Diabetes ☐ Liver disease ☐ Other: ______
21	Do you have first/second degree relatives who died after the pandemic?	☐ Yes ☐ No
22	If you have relatives who died after the pandemic, what pathology (disease) did they die from? (If you don't, just write "no"):	Open-ended response

23	Do you think that you have developed long-term effects following the COVID-19 infection, or do you have a close relative in this situation? If so, please describe these effects:	Open-ended response
24	During the pandemic, did you experience an increased level of stress and anxiety?	☐ Yes ☐ No
25	Have you developed or observed symptoms of depression or anxiety among your acquaintances during the pandemic?	☐ Yes ☐ No
26	Have you encountered difficulties in accessing specialized medical services due to the pandemic?	☐ Yes ☐ No
27	Have you noticed a reduction in access to preventive medical services and screenings during the pandemic?	☐ Yes ☐ No
28	Have you been discouraged from seeking medical care for other conditions during the pandemic, which could affect life expectancy?	☐ Yes ☐ No
29	Have you felt a decrease in the level of trust in the health system following the pandemic, which could affect life expectancy?	☐ Yes ☐ No
30	What sources of health information did you seek or prefer during the pandemic?	☐ Doctors ☐ Internet ☐ Relatives ☐ Friends ☐ Books ☐ Other: _____
31	Do you believe that vaccination against COVID-19 is an effective solution for reducing the risk of getting sick?	☐ Yes ☐ No
32	Do you think that life expectancy has decreased in Romania after the COVID-19 pandemic?	☐ Yes ☐ No
33	Do you think that the decrease in life expectancy is related to a higher incidence of chronic diseases following the pandemic?	☐ Yes ☐ No
34	Do you think the pandemic has led to greater individual responsibility for your health, which could contribute to increasing life expectancy?	☐ Yes ☐ No
35	In your opinion, what factors can contribute to increasing life expectancy?	☐ Adopting a healthy lifestyle ☐ Reducing stress ☐ Improving access to healthcare ☐ Developing medical technology ☐ Reducing pollution and exposure to toxic substances ☐ Adequate rest ☐ Other: ______

36	Do you think the pandemic has led to an increase in interest in epidemiological and public health research?	☐ Yes ☐ No
37	Were you more interested in learning about disease prevention strategies and technologies during the pandemic?	☐ Yes ☐ No

Supplementary File S1. Full Questionnaire Used in the Study: Health Perceptions and Trust in Healthcare after COVID-19 (Romania, 2025)
