# Supplementary material for: Health Perceptions and Trust in Healthcare After COVID-19: An Exploratory Cross-Sectional Survey from Romania
Source: Int J Environ Res Public Health. 2025 Sep 27;22(10):1496. doi: 10.3390/ijerph22101496 (PMC12564094; doi:10.3390/ijerph22101496)
Supplement: Supplementary file 1 [file ijerph-22-01496-s001.zip › ijerph-3809423_Supplementary Table S1.pdf]

		P value
Gender	Medical appointment adherence	0.007
	Symptoms of depression/anxiety among private/close people	0.000
	Decreased trust in the healthcare system following the pandemic	0.025
Age groups	Healthy diet	0.000
	Increased stress & anxiety during pandemic	0.000
Residence	Postponement/avoidance of medical examinations during the pandemic	0.006
	Perceived decrease in LE following the pandemic	0.000
	Increased interest in public health and epidemiological research	0.000
Education level	Difficulty accessing specialist/primary care	0.000
	Reduced access to preventive care	0.000
	Increased stress & anxiety during the pandemic	0.000
	Perceived decrease in LE following the pandemic	0.000

Supplementary Table S1. Statistically significant associations between demographic characteristics and pandemic-related outcomes
