# Supplementary figures and images for: Health Perceptions and Trust in Healthcare After COVID-19: An Exploratory Cross-Sectional Survey from Romania
Source: Int J Environ Res Public Health. 2025 Sep 27;22(10):1496. doi: 10.3390/ijerph22101496 (PMC12564094; doi:10.3390/ijerph22101496)

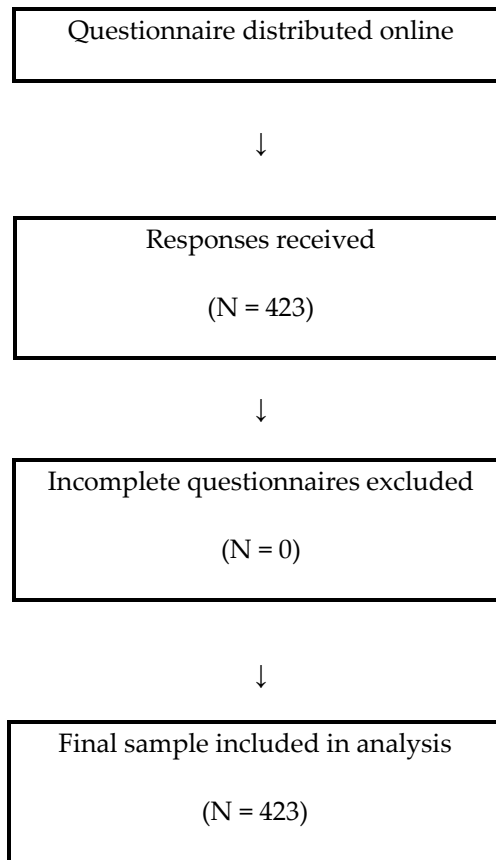

Supplementary Figure S1. Flowchart of inclusion process.

Supplement: Supplementary file 1 [file ijerph-22-01496-s001.zip › ijerph-3809423_Supplementary Figure S1.pdf]
